# Uncovering association networks through an eQTL analysis involving human miRNAs and lincRNAs

**DOI:** 10.1038/s41598-018-33420-z

**Published:** 2018-10-09

**Authors:** Paulo R. Branco, Gilderlanio S. de Araújo, Júnior Barrera, Guilherme Suarez-Kurtz, Sandro José de Souza

**Affiliations:** 1 Bioinformatics Multidisciplinary Environment (BioME), Instituto Metrópole Digital, UFRN, Natal, Brazil; 2 Ph.D Program in Bioinformatics, Instituto Metrópole Digital, UFRN, Natal, Brazil; 3 Instituto de Matemática e Estatística, Universidade de São Paulo, São Paulo, Brazil; 4 Instituto Nacional do Câncer, Rio de Janeiro, Brazil; 5 Instituto do Cérebro, UFRN, Natal, Brazil

## Abstract

Non-coding RNAs (ncRNA) have an essential role in the complex landscape of human genetic regulatory networks. One area that is poorly explored is the effect of genetic variations on the interaction between ncRNA and their targets. By integrating a significant amount of public data, the present study cataloged the vast landscape of the regulatory effect of microRNAs (miRNA) and long intergenic noncoding RNAs (lincRNA) in the human genome. An expression quantitative trait loci (eQTL) analysis was used to identify genetic variants associated with miRNA and lincRNA and whose genotypes affect gene expression. Association networks were built for eQTL associated to traits of clinical and/or pharmacological relevance.

## Introduction

Non-coding RNAs (ncRNAs) are essential components of the vast landscape of human genetic regulatory networks. Among ncRNAs, two types have been shown to be important regulators of gene expression: microRNAs (miRNAs) and long intergenic noncoding RNAs (lincRNAs). miRNA are small ncRNAs of approximately 22 nucleotides produced by two RNase III proteins, Drosha and Dicer^1^. They interact with specific binding sites in mRNAs and regulate gene expression through mRNA degradation and consequently translational repression^1^. Studies on miRNAs are also becoming fundamental for a better understanding of the physiological processes associated with complex diseases^2–5^. miRNAs also have crucial roles in the development and metabolism of healthy cells, regulating at least 30% of human protein-coding genes^6^. Although less studied than miRNAs, lincRNAs are known to act as decoys, scaffolds, sponges, and guides of protein and RNA molecules in cells, fulfilling essential functions associated with gene expression regulation^7^. Like miRNAs, this class of long ncRNAs has emerged as an important regulator of both normal and pathological states^8,9^.

Although these two classes of ncRNAs have been extensively studied in the last decade, one area that is still little explored is the effect of genetic variations on their functions. The few reports published in this area^10–14^ have stressed out the importance of SNVs and structural variations on ncRNAs and helped to elucidate the genetic basis of complex phenotypes, including the development of diseases.

With this perspective in mind, the present study uses an integrated genome-wide approach to identify genetic variants that overlap with genes coding for lincRNA or miRNA as well as miRNA binding sites. Capitalizing on the availability of large cohorts of individuals with both genome and expression data, an expression Quantitative Trait Loci (eQTL) analysis was performed to measure the putative influence of the genetic alterations on gene expression and identify those eQTL associated to genes of pharmacological and/or clinical relevance. Genome-wide association networks involving eQTL, genes and traits were built, which can be used for the study of complex phenotypes in humans.

## Materials and Methods

**Figure 1:**
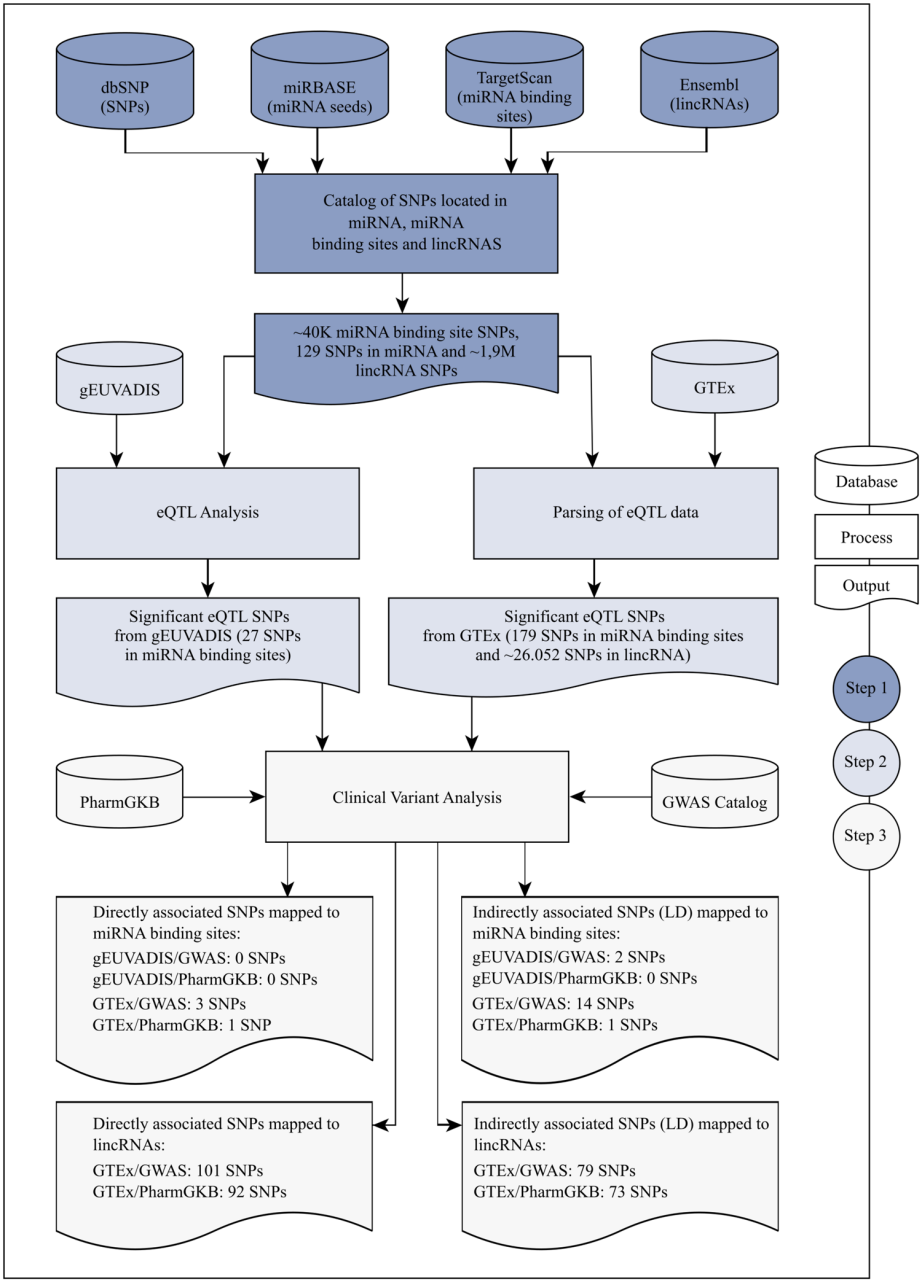
Schematic representation of the analysis workflow for the present study. In Step 1, four databases were integrated to identify SNPs mapped to miRNA seeds and miRNA binding sites, as well as SNPs mapped to lincRNAs. Step 2 comprises two processes: an eQTL analysis with gEUVADIS data as well as data extracted from an eQTL analysis from GTEx. Finally, eQTL from both gEUVADIs and GTEx were compared to variant annotation from GWAS Catalog and PharmGKB.

An overview of the whole analysis workflow and data source is depicted in Fig. 1. Our strategy comprises three broad steps: (1) construction of a catalog of SNPs mapped to miRNA seeds, miRNA-binding sites, and lincRNA genes; (2) eQTL analysis of genetic variants and (3) identification of variants possibly associated to either clinical or pharmacological features.

For the first step, the genomic position of each SNP was compared to the genome coordinates of genes coding for miRNAs and lincRNAs as well as with the coordinates of miRNA-binding sites in mRNAs. Data for approximately 153 million SNPs were retrieved from the Single Nucleotide Polymorphism Database (dbSNP), a public-domain archive for a large collection of single genetic polymorphisms (https://www.ncbi.nlm.nih.gov/snp/), release 150. SNPs were then mapped to almost 6,000 miRNA families that were obtained from the release 21 of the miRBase dataset (http://www.mirbase.org)^15–19^, while TargetScan, release 7.1, was used to retrieve the position of miRNA-binding sites in mRNAs, which resulted in over 123,000 sites predicted^20^. Genome positions for 4,519 lincRNAs were retrieved from release 89 of Ensembl (http://grch37.ensembl.org/index.html). Based on the size distribution of annotated lincRNAs (Ensembl), a threshold was defined to exclude extremely long lincRNAs. Only SNPs mapped to lincRNAs longer than 200 bp and shorter than 100Kbp were used in the subsequent analysis. This filter generated a set of 4,059 lincRNAs and ~1,9 million mapped SNPs.

In the second step of the workflow shown in Fig. 1, an eQTL analysis was performed to measure the contribution of cataloged SNP in the expression level of genes. RNA-seq data for 373 Europeans - CEPH (CEU), Finns (FIN), British (GBR), Toscani (TSI) - were extracted from gEUVADIS (Genetic European Variation in Health and Disease) (http://www.geuvadis.org/web/geuvadis)^21^. The individuals in gEUVADIS are a subset of the individuals available from the 1000 Genomes Project^22^. A Spearman correlation coefficient test was used to evaluate any putative association between genotype and gene expression. An adjusted p-value was generated after correction for multiple testing using the Benjamini-Hochberg method (using a threshold of 0.05 for the adjusted p-value). Additionally, we integrated the Genotype-Tissue Expression (GTEx) Project (version 6) that provides correlations between genotypes and tissue-specific gene expression (https://www.gtexportal.org/home/). Over 2 million GTEX eQTL grouped by 44 tissues were extracted from the project repository^23^ and compared to the cataloged SNPs.

In the third step of our workflow (Fig. 1), eQTL from both gEUVADIS and GTEx analyses were compared to data from the NGHRI/EBI GWAS Catalog^24^ (release 1.0.1) and from PharmGKB^25^ (data download in September 2017) to select eQTL associated to genes of clinical and/or pharmacological interest.

**Figure 2:**
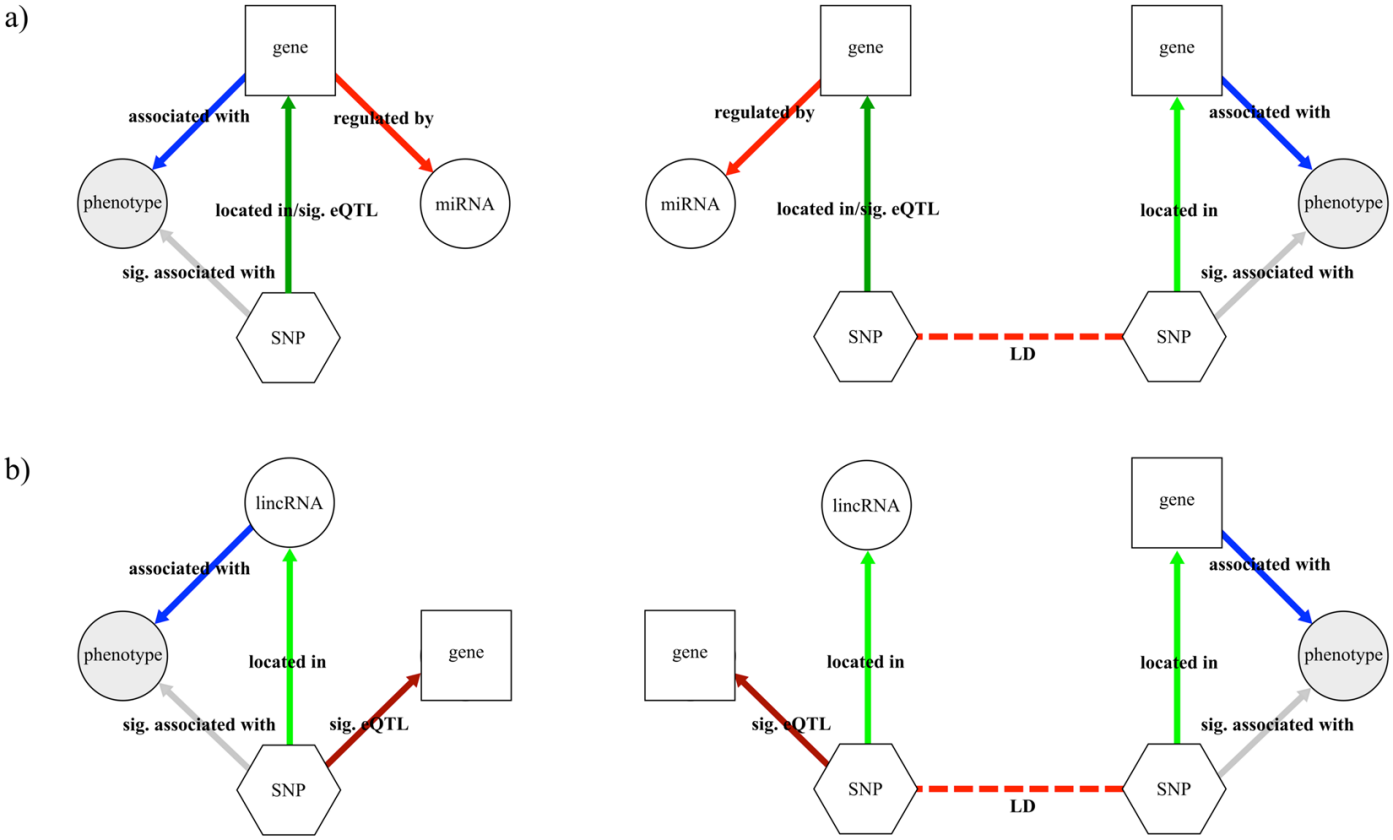
A graphical model for miRNA eQTL association networks. (**a**) Graphical model for miRNA eQTL association networks for direct analysis (left) and indirect analysis as defined by linkage disequilibrium (right). (**b**) Graphical model for lincRNA eQTL association networks for direct analysis (left) and indirect analysis by linkage disequilibrium (right). The elements of the network were represented by distinct shapes, as follows: genes as squares, SNPs as hexagons, lincRNAs and miRNAs as white circles and phenotypes as grey circles. Directed edges (arrows) are represented as follows: genes regulated by miRNAs as light red, SNPs located in genes as light green, eQTL associations as dark red, SNPs located and eQTL associated with genes as dark green, SNPs associated with phenotypes as grey and genes or lincRNAs associated with phenotypes as blue. Undirected edges (light red dashed lines) represent SNPs in high linkage disequilibrium.

The clinically-relevant eQTL were used to model two association networks (illustrated in Fig. 2), as defined below:

- The miRNA association network is a multi-graph *G* = (*V*, *E*) (Fig. 2a), in which the node set *V* comprises four disjoint subsets of SNPs, genes, miRNA, and phenotypes, and the edge set *E* can be decomposed in five node relationships, such as “*located in/sig*. *eQTL*” that links a SNP located in a miRNA binding site to a gene; “*located in*” links a SNP that is located in a gene; “*sig*. *associated with*” links SNPs with phenotypes, if there is an genetic association reported on GWAS Catalog; “*associated with*” links a gene and a phenotype also based on the associations reported by the GWAS Catalog; “*regulated by*” links a gene to a miRNA based on the TargetScan binding sites prediction; and “*LD*” links two SNPs if the index of linkage disequilibrium (R^2^) is greater than or equal to 0.8.
- The lincRNA association network also is a multi-graph *G* = (*V*, *E*) (Fig. 2b), in which the node set *V* comprises four disjoint subsets of SNPs, genes, lincRNAs, and phenotypes. The edge set *E* can be decomposed in five node relationships, such as “*located in*” that links a SNP located in a lincRNA; “*sig*. *associated with*” that links a SNP with a given phenotype if there is an association reported in GWAS catalog; “*associated with*” links a gene and a phenotype also based in a given association reported by GWAS Catalog; “*sig*. *eQTL*” links a SNP to a gene if there is an eQTL reported in GTEx; and “*LD*” links two SNPs if the index of linkage disequilibrium (R^2^) is greater than or equal to 0.8. Since there is no predicted gene targets for lincRNAs, a direct association between lincRNA and a given gene could not be established (as we have done for miRNAs).

The adjacency lists were loaded in Cytoscape v3 for graphical representations and layout editing. All Python scripts developed here are available at https://github.com/paulorobertobranco/Uncovering-association-networks-through-an-eQTL-analysis-involving-human-miRNAs-and-lincRNAs.

## Results and Discussion

### Catalog of SNPs mapped to lincRNA, miRNA seeds and miRNA-binding sites

SNPs were mapped to miRNA seed regions, to their putative binding sites in all human mRNAs and to lincRNA-coding genomic regions to identify genetic variants that could affect gene expression. By integrating dbSNP, miRBase, TargetScan and Ensembl (Fig. 1), our method identified 40,009 SNPs located in miRNA binding sites (Supplementary Table S1 for a complete list of all SNPs), 129 SNPs in miRNA seed regions (Supplementary Table S2) and 1,964,426 SNPs in lincRNAs-coding genomic regions (Supplementary Table S3).

Enrichment analysis using data from the Kyoto Encyclopedia of Genes and Genomes (KEGG) was performed to identify, in the set of genes where the mapped SNPs were located, any possible enrichment for biological pathways. Among the most significant (p-adjusted < = 0.05) enriched pathways, associations with some disease-related pathways, such as cancer, diabetes, and depression were found (see Supplementary Fig. S1).

### eQTL analysis of gEUVADIS data and tissue-specific eQTL from GTEx

**Figure 3:**
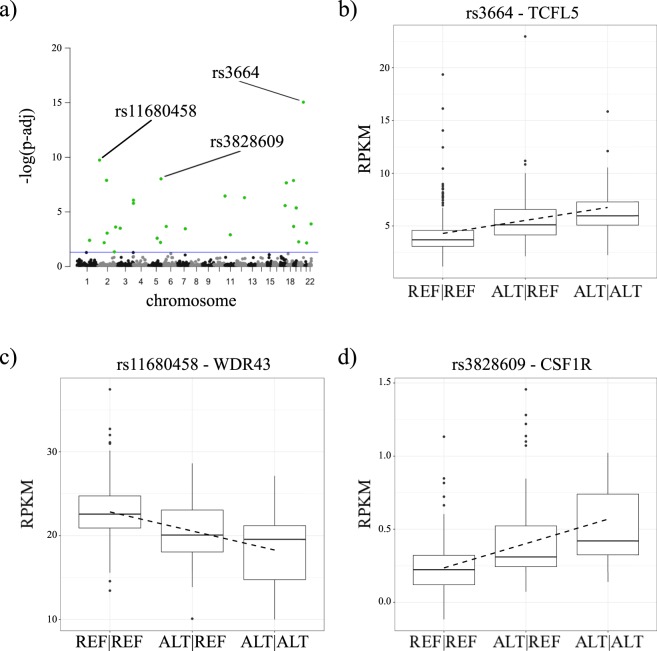
gEUVADIS eQTL analysis results. (**a**) Manhattan plot derived from gEUVADIS eQTL mapped to miRNA-binding sites. Significant SNPs are identified by green dots and threshold (p-adj < = 0.05) is represented by the blue line. (**b**) Boxplot of TCFL5 expression level (as measured by RPKM) in gEUVADIS samples grouped by rs3664 genotype. (**c**) Boxplot of WDR43 expression level in gEUVADIS samples grouped by rs11680458 genotype. (**d**) Boxplot of CSF1R expression level on gEUVADIS samples grouped by rs3828609 genotype.

Next, we investigated whether genetic variants in miRNA seeds, miRNA-binding sites and lincRNAs-coding genomic regions could affect gene expression. Transcriptome and genome data available from gEUVADIS were used to perform an eQTL analysis, as described in Material and Methods. As a result, a set of 27 SNPs in miRNA binding sites were found to be significantly associated with the expression of the corresponding genes (genes where the respective miRNA-binding site was present). A Manhattan plot resulting from the eQTL analysis can be seen in Fig. 3a and details of all 27 significant SNPs are shown in Supplementary Table S4. The three most significant eQTL were: rs3664 (p-adjusted = 9.1e–16, correlation coefficient = 0.44) that may affect the binding between miR-30-5p (a tumor suppressor miRNA) and transcripts from TCFL5 (Fig. 3b); rs11680458 (p-adjusted = 1.8e–10, correlation coefficient = 0.37) possibly affecting the binding between miR-141-3p and WDR43 (Fig. 3c); and rs3828609 (p-adjusted = 9.5e–09, correlation coefficient = 0.34) that may affect the affinity between miR-155-5p and CSF1R (Fig. 3d). TCFL is a transcription factor whose expression seems to be a prognostic factor for childhood acute lymphoblastic leukemia^26^. miR-141-3p is also a cancer-related miRNA acting either as a tumor suppressor^27,28^ or an oncogene^29^, and WDR43 has been recently associated with the etiology of estrogen receptor (ER)-negative breast cancer^30^. CSF1R has been associated with several hematological-related traits^31^, while a relationship between miR-155-5p and papillary thyroid carcinoma diagnosis was described by Jahanbani *et al*.^32^.

No significant eQTL was found in the analysis involving SNPs mapped to miRNA seeds. On the other hand, the large number of SNPs mapped to lincRNA regions made the eQTL analysis computationally intractable, since a whole-genome eQTL analysis should be calculated by each one of the ~1,9 M SNPs. This limitation of the present work is being considered for future studies.

**Figure 4:**
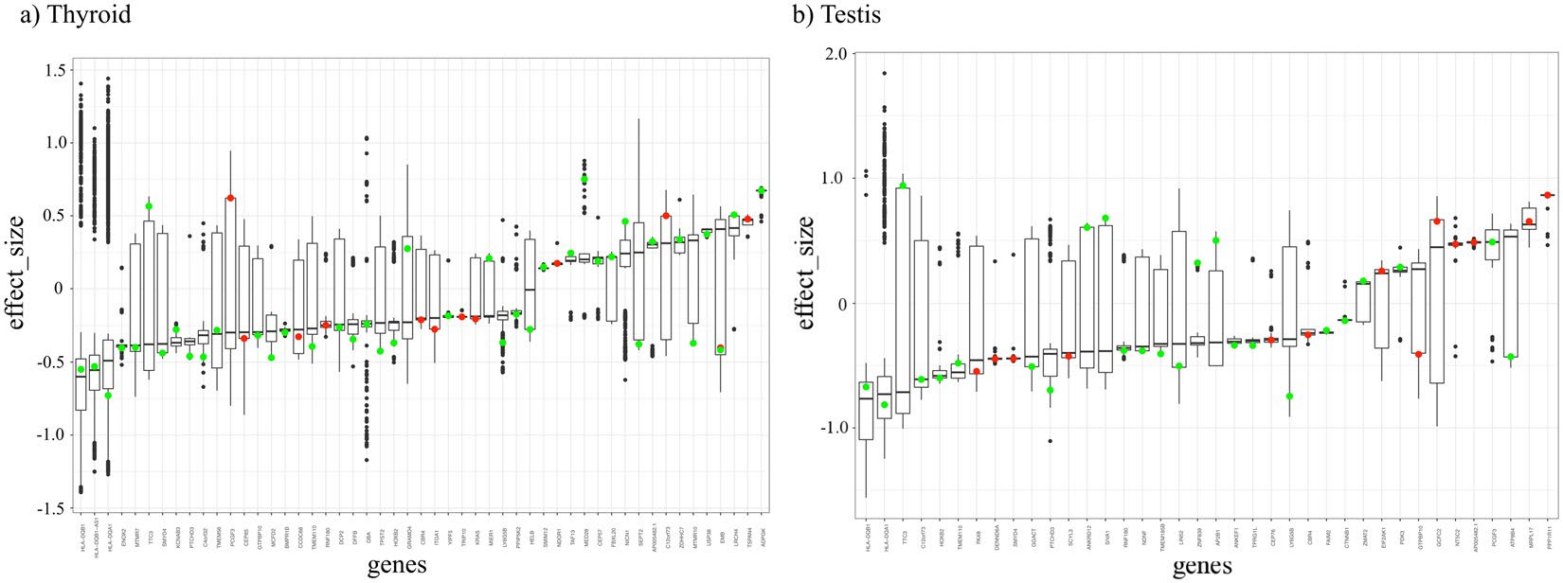
Distribution of effect size for significant eQTL (derived from GTEx). Y-axis represents the putative SNP effect size (slope) over gene expression. X-axis represents the SNPs grouped by the gene where they are located. SNPs mapped to miRNA binding sites are represented by red or green dots for thyroid (**a**) and testis (**b**) tissues. Green dots represent those SNPs that are outliers for the respective distribution (z-score >2 or z-score <−2).

Using the available eQTL data from GTEx, our strategy identified 180 significant SNPs mapped to miRNA binding sites and 26,052 significant SNPs mapped to lincRNA-coding genomic regions (Supplementary Tables S5 and S6, respectively). No SNP mapped to miRNA seed matched any significant eQTL in the GTEx dataset. Thyroid and testis were the tissues that presented the highest number of e-QTL in both miRNA-binding sites and lincRNAs (Supplementary Fig. S2). By analyzing both tissues, it is possible to notice that some eQTL mapped to miRNA-binding sites diverge from the mean distribution of effect size (Fig. 4). The effect size of a given eQTL is defined as the slope of the linear regression and is computed as the effect of the alternative allele relative to the reference allele (allele reported in the human genome reference sequence). This suggests that these eQTL associated with miRNA-binding sites may have a higher influence on gene expression when compared to eQTL in general. A KEGG enrichment analysis was also performed on such eQTL and returned some common disease-related pathways such as cancer, diabetes, asthma and tuberculosis (Supplementary Fig. S3 for miRNA-binding sites and Supplementary Fig. S4 for lincRNAs).

### Association networks for clinically-relevant eQTL

To assess whether the eQTL identified here could be associated with clinically relevant genes and/or clinically relevant traits, a comparison was made with data from the NGHRI/EBI GWAS Catalog and PharmGKB. Besides a direct comparison, querying the clinical databases with our eQTL, we have also checked whether any of our eQTL were in linkage disequilibrium with any variation present in the clinical databases.

**Figure 5:**
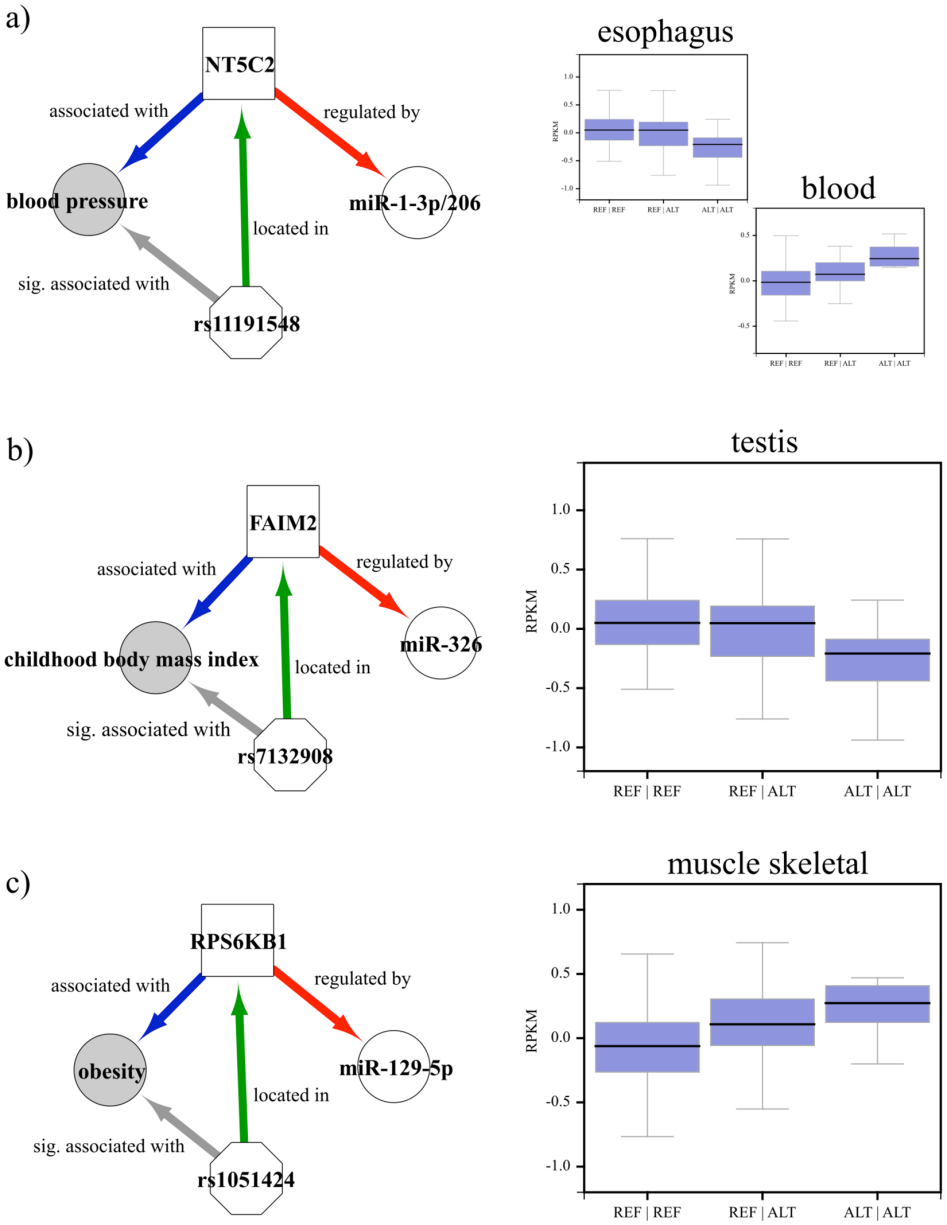
Association networks for eQTL mapped to miRNA-binding sites and present in the GWAS Catalog. (**a**) Association network for rs11191548, located in the binding sites of miR-1-3p and miR-206 in NT5C2 and associated with blood pressure (left). Boxplot of NT5C2 expression levels on GTEx samples (esophagus and blood tissues) grouped by rs11191548 genotype (right). (**b**) Association network for rs7132908, located in the binding site of miR-326 in FAIM2 and associated with the childhood body mass index (left). Boxplot of FAIM2 expression levels on GTEx samples (testis) grouped by rs7132908 genotype (right). (**c**) Association network for rs1051424, located in the binding site of miR-129-5p in RPS6KB1 and associated with obesity (left). Boxplot of RPS6KB1 expression levels on GTEx samples skeletal (muscle) grouped by rs1051424 genotype (right).

To simplify the interpretation of the resulting data, eQTL identified using the gEUVADIS and GTEx data were pooled together. Clinically relevant eQTL were used to build association networks involving genes and traits linked to a given eQTL. Direct comparison of eQTL associated with miRNA binding sites and the clinical databases identified three variations associated with GWAS studies (see Fig. 5 and Supplementary Table S7): (i) rs1051424 that affects the expression of RPS6KB1 (p-adjusted = 2.2e–05) in skeletal muscle tissue and has been associated to obesity-related traits^33^; (ii) rs11191548 that affects the expression of NT5C2 in two different tissues (esophagus and blood with p-adjusted equals to 3.6e–05 and 5.6e–05, respectively), and has been associated to blood pressure in four different studies^34–37^ and finally, (iii) rs7132908 that affects the expression of FAIM2 (p-adjusted = 3.0e–05) in testis, and has been associated to childhood body mass^38^. To provide a better overview of the associations, networks for all three individual eQTL, as well as their distribution of expression level according to the respective genotypes, are seen in Fig. 5.

Our workflow identified one miRNA-binding site eQTL in the PharmGKB database. SNP rs712 is located in the target site of miR-877-5p and may affect the expression of an important pharmacogene, KRAS. Besides its involvement in several normal physiological processes, KRAS is related to response to cetuximab and panitumumab, two epidermal growth factor receptor (EGFR) inhibitor drugs used in the treatment of some types of cancer. Additionally, LD analysis reported that rs712 is in high linkage disequilibrium (R^2^ > = 0.8) with a set of 35 SNPs (Supplementary Table S8), also located in KRAS.

LD analysis identified 16 pairs of miRNA-binding sites eQTL showing an LD relationship with a variant present in the GWAS Catalog repository. Two of the most interesting ones are: (a) rs6664467 (mapped to gene MRPL9-TDRKH) is associated with carotid plaque burden^39^ in GWAS Catalog and is in high LD (R^2^ = 0.85) with rs6683364, an eQTL (p-adjusted = 0.004) identified here; (b) the second pair is rs11680458 and rs67073037 with high linkage disequilibrium (R^2^ = 0.97). The eQTL rs11680458 possibly affects the expression of WDR43 (p-adjusted = 1.8e–10) and rs67073037 was recently associated with breast cancer in GWAS Catalog^30^. For more details on the additional eQTL, please see Supplementary Table S9.

A comparison of eQTL mapped to lincRNAs and present in NGHRI/EBI GWAS Catalog identified 101 SNPs directly associated with GWAS studies (Supplementary Table S10). Interestingly, 11 of them were related to 7 different types of cancer: breast cancer (rs2016394), colorectal cancer (rs1372474, rs2293582), endometrial cancer (rs13328298, rs1777220, rs2797160), Ewing sarcoma (rs4924410), Hodgkin’s lymphoma (rs1432295), prostate cancer (rs11672691, rs8014671), and testicular germ cell cancer (rs4561483). Besides cancer, some other disease-related phenotypes were also reported, such as allergy, asthma, blood pressure, hepatitis C and HIV, and neuropsychiatric traits, such as schizophrenia, bipolar disorder, migraine and response to antipsychotic treatments.

A comparison of eQTL mapped to lincRNAs and present in PharmGKB database analysis resulted in 92 SNPs (Supplementary Table S11) that may affect the expression of some critical pharmacogenes, including *BRCA1*, *CYP2D6*, *CYP4F2*, *DPYD*, *DRD2*, *HLA-B*. Two eQTL (rs7223460, rs11396510) were negatively associated with the expression of BRCA1. *CYP2D6* gene is associated with response to medications used to treat a number of mental illnesses (depression, anxiety disorder, attention deficit hyperactivity disorder and bipolar disorder), heart-related diseases (congestive heart failure, left ventricular dysfunction and high blood pressure), allergic conditions (rhinitis and urticaria) and others. Fifteen significant SNPs that regulate *CYP2D6* expression levels were reported. Three eQTL influence the expression of *CYP4F2*, a gene related to blood pressure diseases and associated with anticoagulant drugs, such as warfarin. rs5776391 and rs2000920 may significantly influence the expression of *DPYD* and *DRD2* genes, respectively. *DPYD* is associated with response to capecitabine, fluorouracil and tegafur, all of them used to treat different types of cancer. *DRD2* is associated with antipsychotic medications used to manage schizophrenia, bipolar disorder and related diseases. At least, 70 SNPs related to *HLA-B* gene were reported. They were associated with 12 drugs, such as abacavir, used to prevent and treat HIV/AIDS.

Indirectly, 79 lincRNA eQTL are in high linkage disequilibrium (R^2^ > = 0.8) with 80 GWAS associated SNPs (Supplementary Table S12). From this analysis, disease-related phenotypes such as asthma, bipolar disorder, blood pressure, colorectal cancer, depression or major depressive disorder and endometrial cancer were observed. Also, indirectly, 73 SNPs mapped to lincRNA are in high LD (R^2^ > = 0.8) with 72 eQTL (Supplementary Table S13) that may regulate some important pharmacogenes, including *BRCA1*, *CYP2D6*, *CYP4F2* and *HLA-B*.

**Figure 6:**
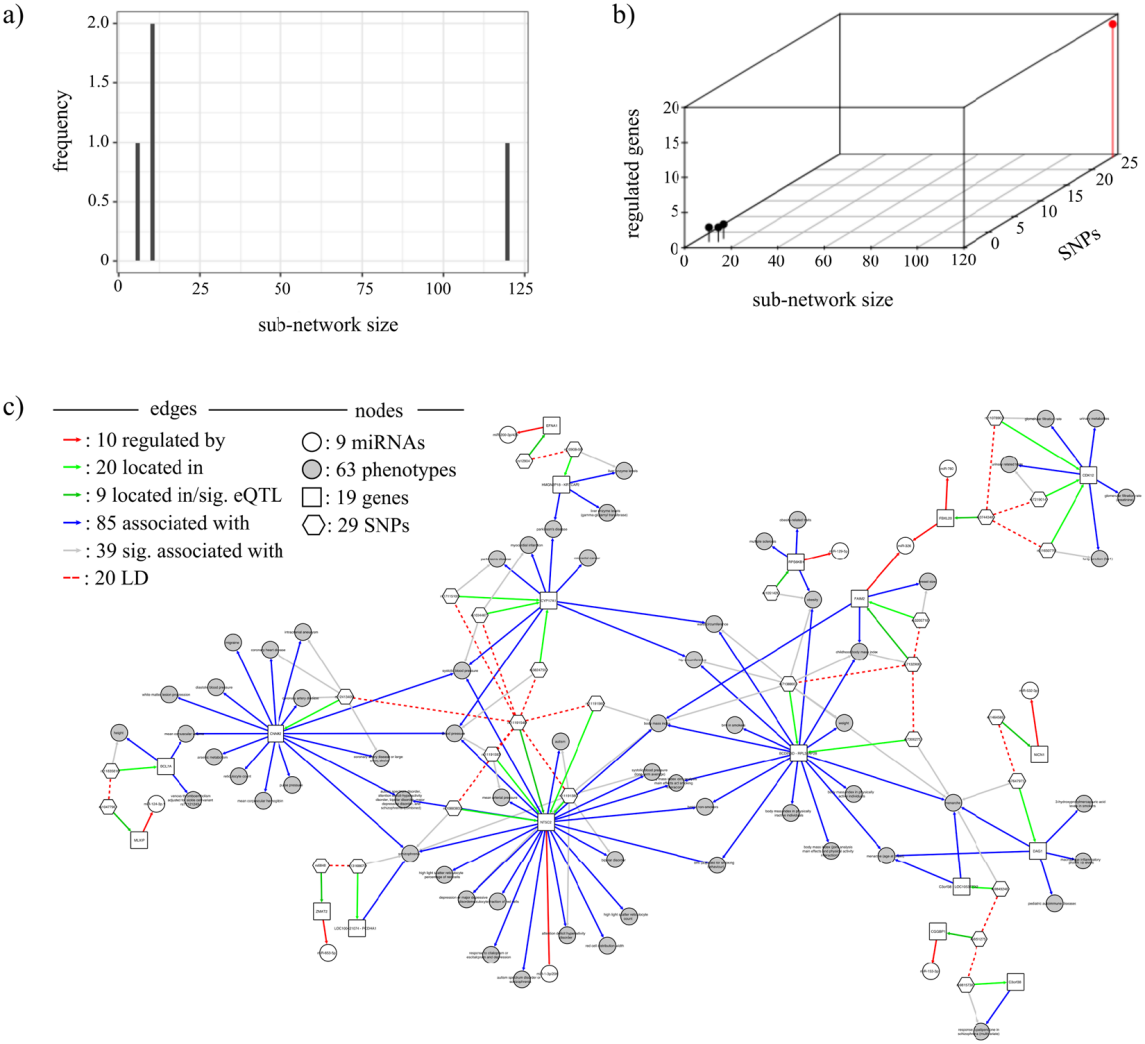
Association network built using all GWAS-linked eQTL mapping to miRNA-binding sites. (**a**) Sub-network distribution size for the association network. (**b**) Distribution of the number of regulated genes and the number of SNPs based on the sub-network size. The largest sub-network is shown in red. (**c**) Graphical representation of the largest sub-network identified by the red bin in (**b**).

**Figure 7:**
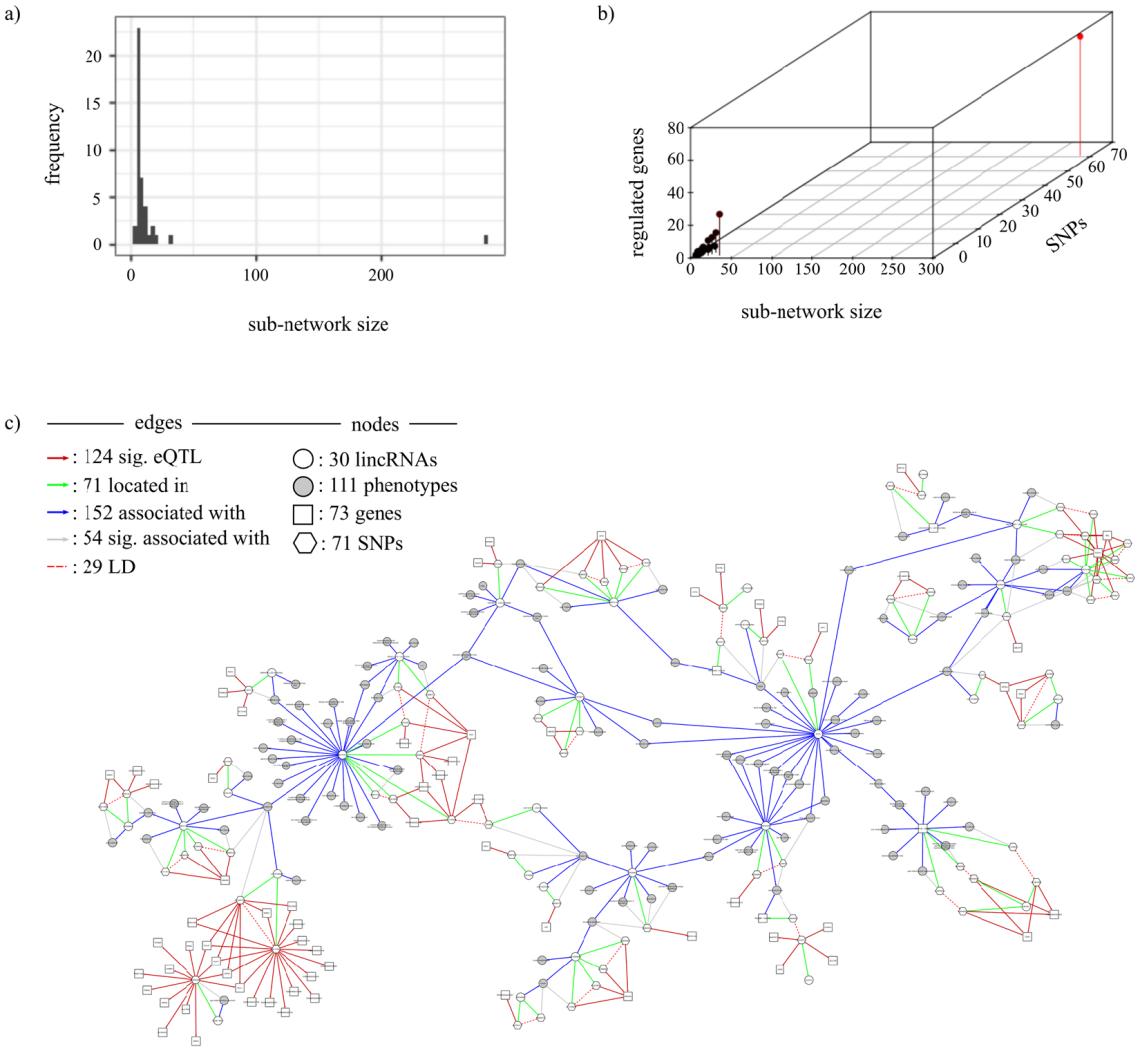
Association network built using all GWAS-linked eQTL mapping to lincRNA. (**a**) Sub-network distribution size for the network. The X-axis represents the component size and Y-axis represents the frequency of the component. (**b**) Distribution of regulated genes based on the sub-network size and the number of SNPs. The X-axis represents the sub-network size, Z-axis represents the number of SNPs and Y-axis represents the number of regulated genes. Largest sub-network is shown in red. (**c**) Graphical representation of the largest sub-network identified by the red bin on (**b**).

To uncover any other possible relationship between clinically relevant eQTL, we decided to model two association networks including all respective GWAS-linked eQTL, one for miRNA and the other for lincRNA. The association network for miRNA presented 146 nodes and 2010 edges distributed in four sub-networks (Fig. 6a,b). The largest sub-network is shown in Fig. 6c, while the remaining sub-networks are shown in Supplementary Fig. S5. The association network for lincRNA was more fragmented with 42 sub-networks comprising 629 nodes and 887 edges (Fig. 7a,b). The largest sub-network is shown in Fig. 7c while all the remaining sub-networks are shown in Supplementary Fig. S6.

**Figure 8:**
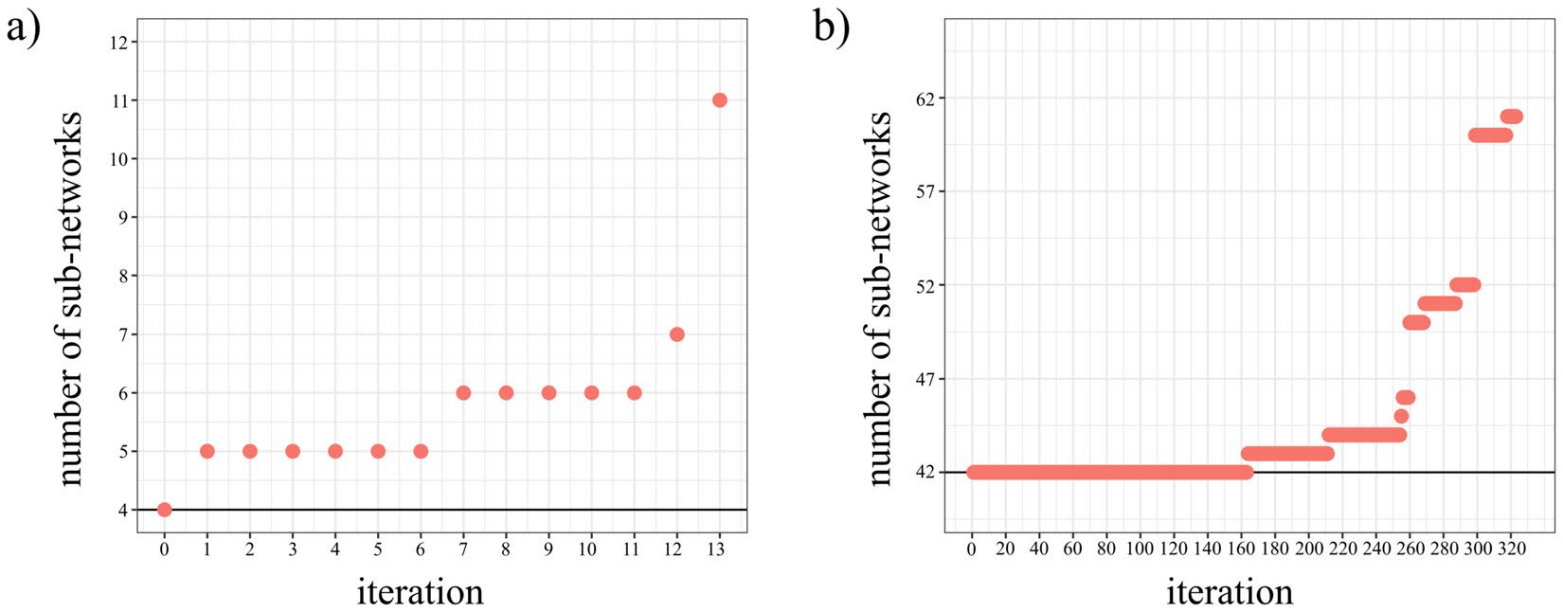
Impact of the removal of individual eQTL in the topology of association network. Impact on miRNA and lincRNA networks is shown in (**a** and **b**), respectively. Iteration zero means the initial state of the network topology. Each subsequent iteration represents the removal of an individual and independent eQTL (X-axis). The number of sub-networks resulted from the respective eQTL removal is shown on the Y-axis.

To evaluate the impact of individual eQTL in the topology of each network, we exhaustively removed all eQTL from the network in an iterative way. For the association network for eQTL mapped to miRNA-binding sites, removal of rs11191548-NT5C2 had the most significant impact in the network increasing the number of sub-networks from 4 to 11 (Fig. 8a). For the association network for eQTL mapped to lincRNA, removal of six eQTL had a similar impact on the network increasing the number of sub-network from 41 to 61 (Fig. 8b). All eQTL involved the SNP rs35181953.

## Conclusions

The approach and data reported here provide a catalog of eQTL mapped to miRNA seeds, miRNA-binding sites and lincRNAs that supposedly affect gene expression of clinical and important pharmacogenes. By taking advantage of heterogeneous biological data sources, as NGHRI/EBI GWAS Catalog and PharmGKB, our workflow and data allow a series of promising new investigations, such as the replication of eQTL analysis in other populations and the study of the selection forces acting on regulatory networks, among others.

## Electronic supplementary material

Supplementary Figures S1-6

Supplementary Tables S1-13
