# Supplementary material for: Uncovering association networks through an eQTL analysis involving human miRNAs and lincRNAs
Source: Sci Rep. 2018 Oct 9;8:15050. doi: 10.1038/s41598-018-33420-z (PMC6177424; doi:10.1038/s41598-018-33420-z)
Supplement: Supplementary file 1 — Supplementary Figures S1-6 [file 41598_2018_33420_MOESM1_ESM.docx]

**Uncovering association networks through an eQTL analysis involving human miRNAs and lincRNAs.**

**Paulo R. Branco^1,2^, Gilderlanio S. de Araújo^1^, Júnior Barrera^3^, Guilherme Suarez-Kurtz^4^ and Sandro José de Souza^1,5,*^**

1. Bioinformatics Multidisciplinary Environment (BioME), Instituto Metrópole Digital, UFRN, Natal, Brasil
2. Ph.D Program in Bioinformatics, Instituto Metrópole Digital, UFRN, Natal, Brasil
3. Instituto de Matemática e Estatística, Universidade de São Paulo, São Paulo, Brasil.
4. Instituto Nacional do Câncer, Rio de Janeiro, Brasil.
5. Instituto do Cérebro, UFRN, Natal, Brasil

* To whom correspondence should be addressed at:

BioME, UFRN

Rua Odilon Gomes de Lima 1722

Capim Macio, Natal, RN

Email: sandro@neuro.ufrn.br

**Supplementary Figures**

**
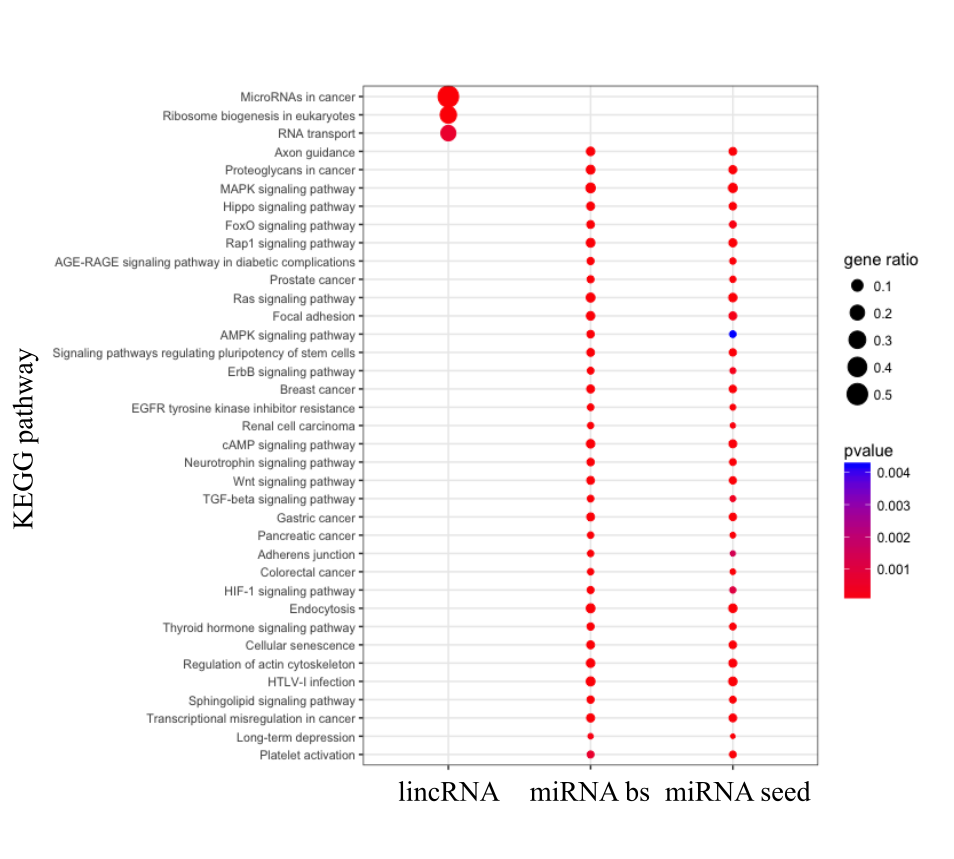
**

**Figure S1.** KEGG enrichment analysis of genes where SNPs mapped to miRNA seeds (right column), miRNA binding sites (middle column) and lincRNAs (left column) are located.

**
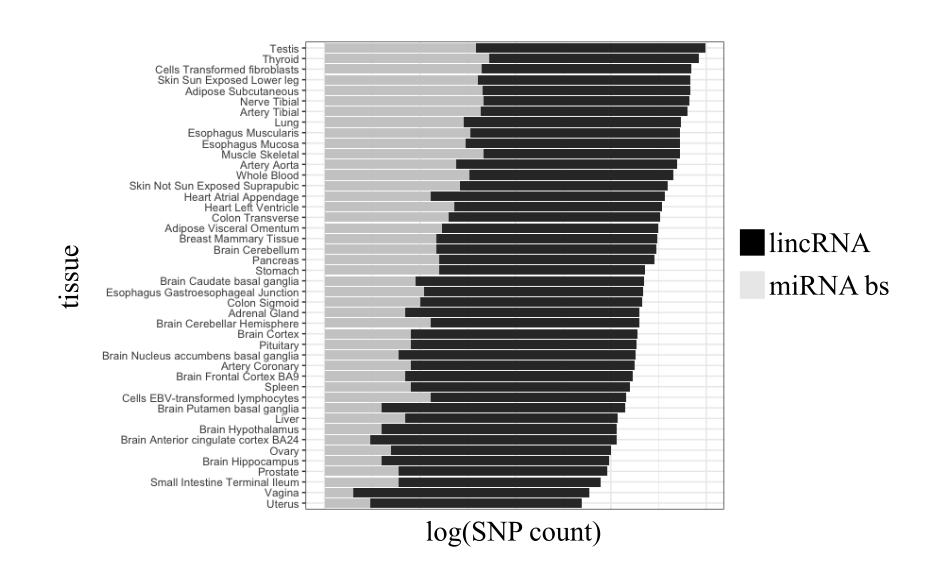
Figure S2.** GTEX eQTL analysis results. The amount of SNPs (log) for each tissue.


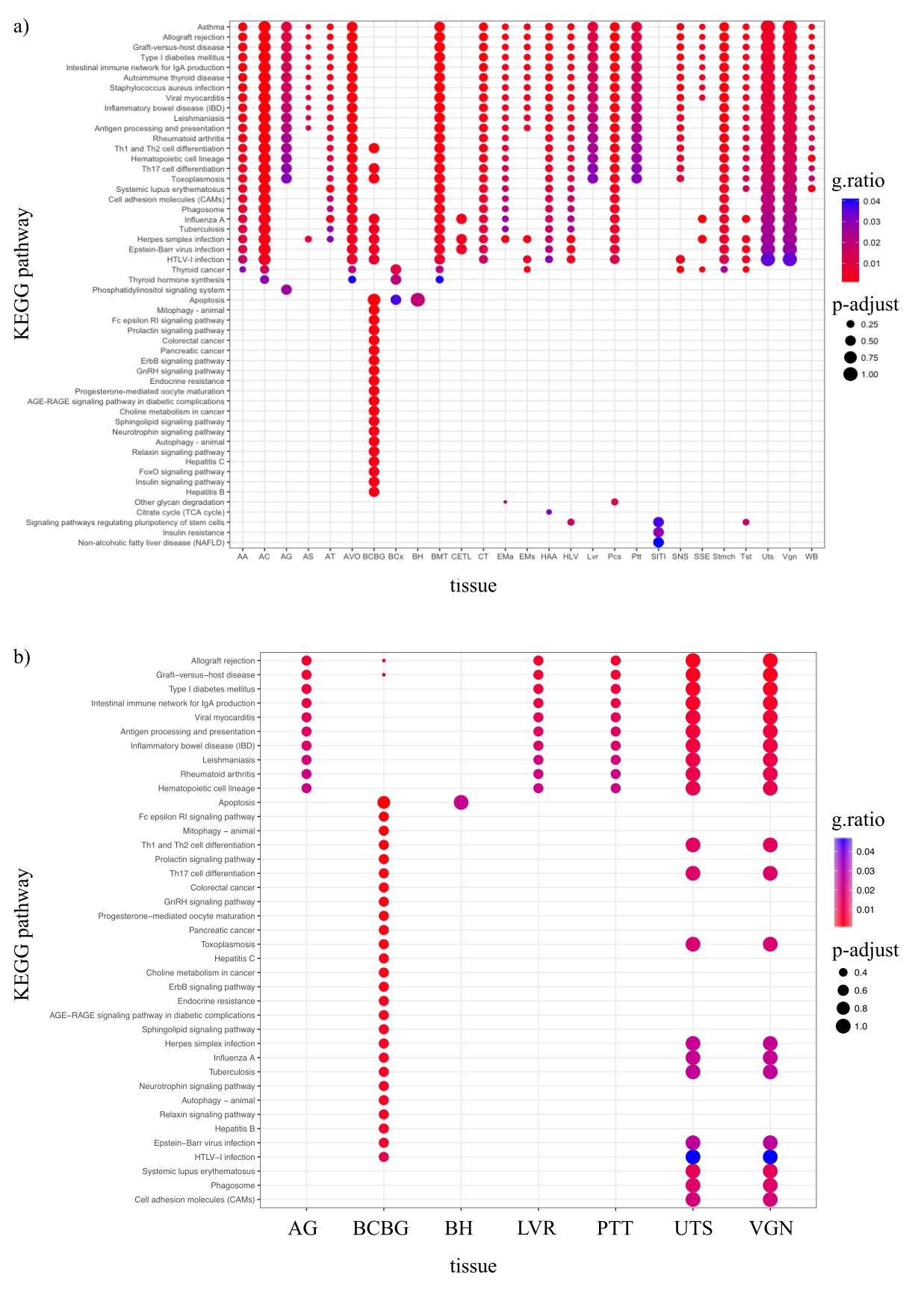
**Figure S3.** KEGG enrichment analysis plot for GTEx eQTL analysis on miRNA binding sites. X-axis represents the tissues and y-axis represents the KEGG pathways. a) All genes are used as the control universe. b) Control universe is composed by genes selected based on all SNPs mapped to the studied regions.


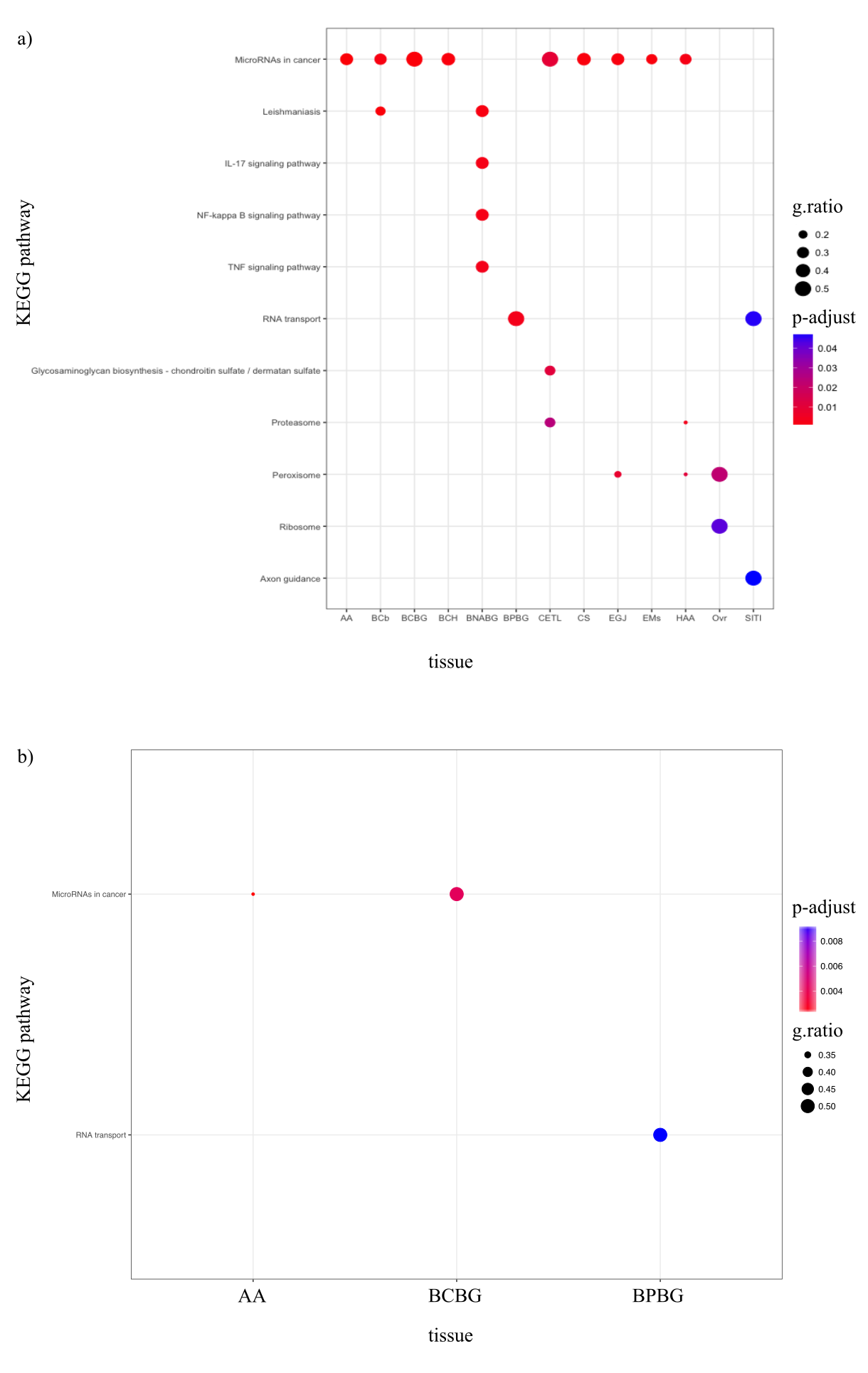
**Figure S4.** KEGG enrichment analysis plot for GTEx overlapping lincRNAs. X-axis represents the tissues and y-axis represents the KEGG pathways. a) All genes are used as the control universe. b) Control universe is composed by genes selected based on all SNPs mapped to the studied regions.

**
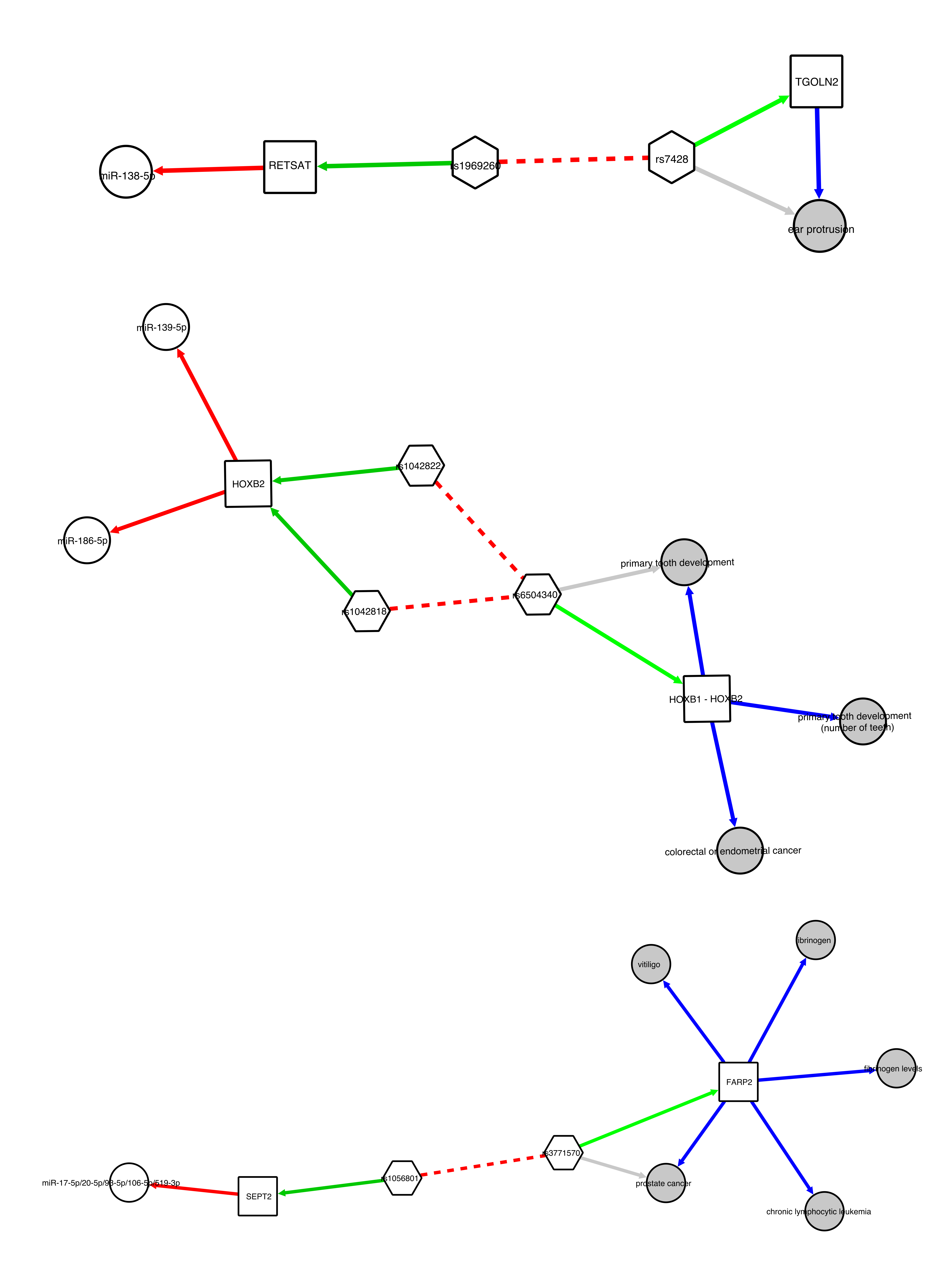
**

**Figure S5.** Smaller miRNA association sub-networks. The largest sub-network is shown as Figure 6C in the main text.


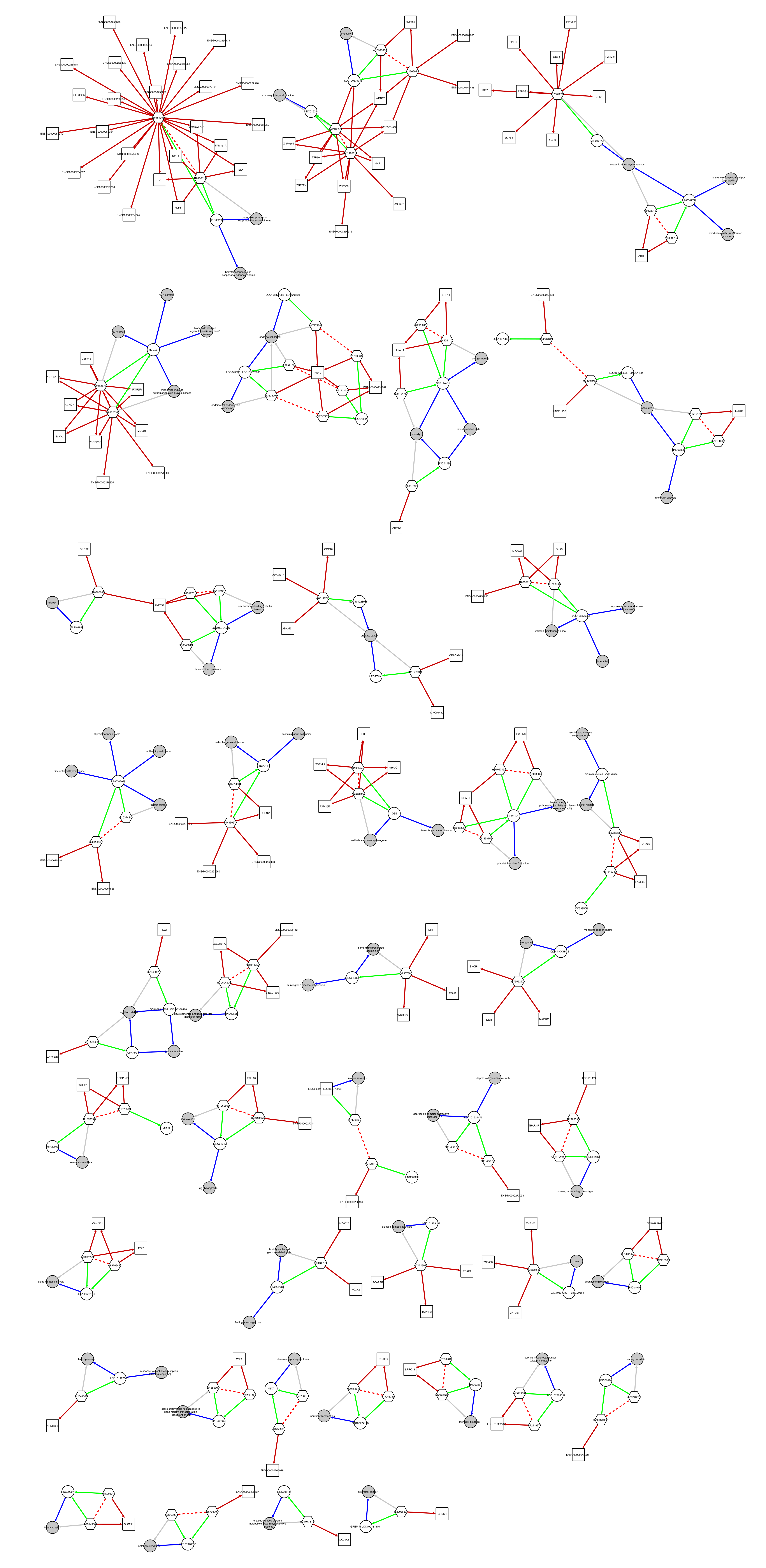


**Figure S6.** Smaller lincRNA association sub-networks. The largest sub-network is shown in Figure 7C in the main text.

**Supplementary Tables**

- Due to file size, Supplementary Table S3 is available at:

<https://github.com/paulorobertobranco/Uncovering-association-networks-through-an-eQTL-analysis-involving-human-miRNAs-and-lincRNAs>. All remaining supplementary tables are available in file “Supplementary Tables. xlsx”. Legends and description for all Supplementary Tables are shown below.

**Supplementary Table S1**

Detailed list of SNPs mapped to miRNA binding sites.

**Supplementary Table S2**

Detailed list of SNPs mapped to miRNA seed regions (CSV - 4 KB).

**Supplementary Table S3**

Detailed list of SNPs mapped to lincRNA.

**Supplementary Table S4**

Detailed list of gEUVADIS significant eQTLs mapped to miRNA binding sites.

**Supplementary Table S5**

Detailed list of GTEx significant eQTLs mapped to miRNA binding sites.

**Supplementary Table S6**

Detailed list of GTEx significant eQTLs mapped to lincRNA.

**Supplementary Table S7**

Detailed list of GTEx significant direct comparison of eQTLs associated with miRNA binding sites and GWAS studies.

**Supplementary Table S8**

Detailed list of GTEx significant indirect comparison of eQTLs associated with miRNA binding sites and PharmGKB.

**Supplementary Table S9**

Detailed list of GTEx significant indirect comparison of eQTLs associated with miRNA binding sites and GWAS studies.

**Supplementary Table S10**

Detailed list of GTEx significant direct comparison of eQTLs associated with lincRNAs and GWAS studies.

**Supplementary Table S11**

Detailed list of GTEx significant direct comparison of eQTLs associated with lincRNAs and PharmGKB.

**Supplementary Table S12**

Detailed list of GTEx significant indirect comparison of eQTLs associated with lincRNAs and GWAS studies.

**Supplementary Table S13**

Detailed list of GTEx significant indirect comparison of eQTLs associated with lincRNAs and PharmGKB.
